# CircRNAs in anticancer drug resistance: recent advances and future potential

**DOI:** 10.1186/s12943-020-01240-3

**Published:** 2020-08-17

**Authors:** Tianwei Xu, Mengwei Wang, Lihua Jiang, Li Ma, Li Wan, Qinnan Chen, Chenchen Wei, Zhaoxia Wang

**Affiliations:** 1 Cancer Medical Center, The Second Affiliated Hospital of Nanjing Medical University, Jiangjiayuan road 121#, Nanjing, 210011 Jiangsu P.R. China; 2 Department of Oncology, The Affiliated Huai’an No.1 People’s Hospital of Nanjing Medical University, Huai’an, 223300 Jiangsu China

**Keywords:** Circular RNA, Cancer, Drug, Resistance

## Abstract

CircRNAs are a novel class of RNA molecules with a unique closed continuous loop structure. CircRNAs are abundant in eukaryotic cells, have unique stability and tissue specificity, and can play a biological regulatory role at various levels, such as transcriptional and posttranscriptional levels. Numerous studies have indicated that circRNAs serve a crucial purpose in cancer biology. CircRNAs regulate tumor behavioral phenotypes such as proliferation and migration through various molecular mechanisms, such as miRNA sponging, transcriptional regulation, and protein interaction. Recently, several reports have demonstrated that they are also deeply involved in resistance to anticancer drugs, from traditional chemotherapeutic drugs to targeted and immunotherapeutic drugs. This review is the first to summarize the latest research on circRNAs in anticancer drug resistance based on drug classification and to discuss their potential clinical applications.

## Introduction

Cancer has become the most serious public health problem worldwide [1]. As the early diagnosis of cancer urgently needs to be improved, many patients are already suffering from advanced cancer at the first clinical visit [2]. These patients miss the opportunity for surgery, and anticancer drugs are their major treatment option. Since the first clinical report of chemotherapy for advanced cancers, multiple anticancer drugs have been developed and have effectively improved the clinical outcomes of patients with advanced cancers [3]. In addition, targeted and immunotherapeutic drugs have recently ushered in a new era in medical therapy for cancer [4, 5]. However, anticancer drug resistance still cannot be avoided, resulting in cancer relapse. The mechanisms underlying anticancer drug resistance are multifaceted. Different hallmarks such as tumor growth, selective therapeutic pressure, immune system characteristics and the tumor microenvironment determine the biological behaviors of anticancer drug resistance [6]. These hallmarks are driven by complex underlying molecular regulatory mechanisms [7]. Identifying the key molecules in these processes could help us to understand the occurrence of resistance, and these molecules play considerable roles in the prediction and reversion of anticancer drug resistance [8].

With the development of sequencing, studies have shown that approximately 70% of the human genome is transcribed, but only 2% encodes proteins [9]. The remaining RNA, which does not encode proteins, was initially thought to be transcriptional junk [10]. Accumulating evidence has revealed that these noncoding RNAs (ncRNAs) also exert a great influence on physiological activities and pathological changes, especially in cancer [11, 12]. Importantly, ncRNAs are classified by size into small RNAs such as miRNAs (~ 22 nt) and long non-coding RNAs (lncRNAs, > 200 nt). Dysregulation of ncRNAs participates in the molecular and cellular processes related to cancer. The roles of ncRNAs are divided into oncogenes and tumor suppressors, based on their effect on the cancer. For example, miR-21 was reported to promote tumor growth and metastasis by targeting PTEN in lung cancer and other cancers, such as melanoma and B cell lymphoma [13, 14]. As shown in our previous study, high serum miR-21 levels indicate a poor prognosis for patients with non-small cell lung cancer (NSCLC) [15]. In contrast, miR-34a functions as a direct downstream target of p53 to block cancer progression [16]. HOTAIR, a well-studied oncogenic lncRNA, is generally upregulated in cancers and epigenetically silences tumor suppressors such as p21 [17]. However, lncRNA MEG3 modulates the expression of p53 and some other tumor suppressors to increases chemotherapeutic sensitivity [18]. Several ncRNAs may also function as either tumor suppressors or oncogenes, depending on the context. For example, the lncRNA NKILA reduces cancer metastasis by negatively regulating NF-kB signaling, while it also promotes tumor immune evasion [19]. Since ncRNAs such as miRNAs and lncRNAs have been confirmed to play an important role in cancer, studies of new ncRNAs will continue to improve our understanding of cancer.

**Fig. 1:**
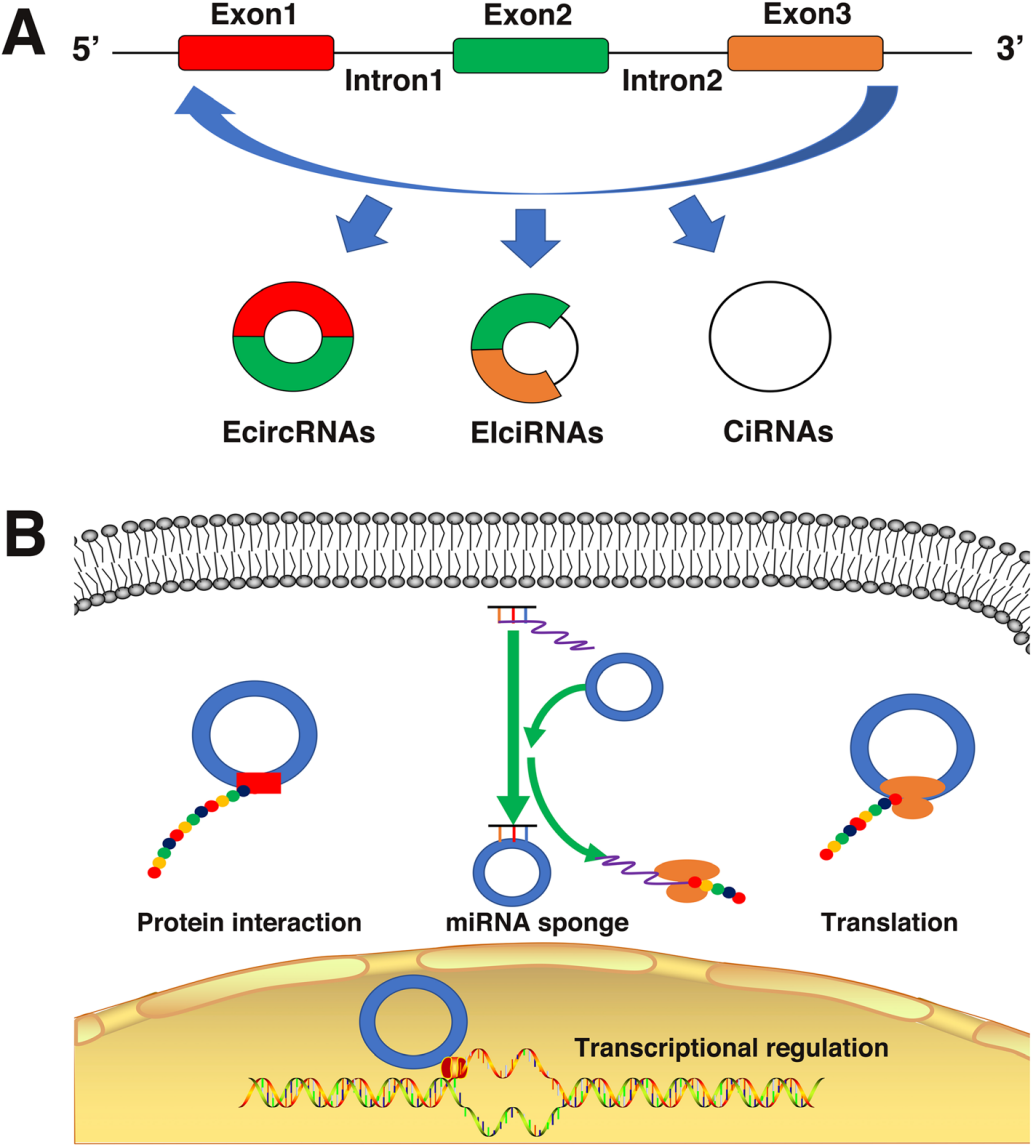
Biogenesis of circRNAs. **a** Classification of circRNAs. CircRNAs can be divided into ecircRNAs, EIciRNAs and ciRNAs by their composition. **b** Functions of circRNAs. CircRNAs can regulate gene transcription as their parental genes do in the nucleus. CircRNAs can act as miRNA sponges, interact with proteins as protein scaffolds or decoys and, under certain circumstances, be translated in the cytoplasm

In recent years, a novel class of lncRNAs designated circular RNAs (circRNAs) has also been identified and extensively studied in cancer biology [20]. CircRNAs are derived from pre-mRNAs through a non-canonical alternative splicing event called backsplicing. This process endows circRNAs with a unique fundamental structural feature that differs from common linear lncRNAs, namely, a covalently closed continuous loop structure without a polyadenylated tail. CircRNAs are very stable because their unique structure is resistant to exonuclease-mediated degradation [21]. Although backsplicing is much less efficient than canonical splicing in linear RNAs, circRNAs are still enriched in tissues, serum and even urine [22]. Although circRNAs are widely expressed, the expression pattern of circRNAs displays tissue specificity and cell type specificity. For example, platelets express more circRNAs than neutrophils, which are also blood cells (3324 vs 274 circRNAs) [23, 24].CircRNAs can be divided into 3 subtypes: 1) exonic circRNAs (EcircRNAs), which contain only exon sequences; 2) intronic circRNAs (ciRNAs), which contain only intron sequences; and 3) exon-intron circRNAs (EIciRNAs), which contain both exon and intron sequences [25] (Fig. 1a). Studies have demonstrated that circRNAs may exert biological functions by transcriptional regulation, miRNA sponging, protein interactions or, under certain circumstances, self-translation [26, 27] (Fig. 1b). For example, ciRS-7 is a well-known circRNA that is enriched in neuronal tissues. CiRS-7 knockout mice exhibit neuropsychiatric disorders, and the underlying mechanism may be attributed to the ciRS-7/miR-7/Fos axis [28].

CircRNAs also participate in various pathological processes, including the development of cancer [22]. According to the classification of the mechanism in tumors, we present some representative circRNAs with clear mechanisms, biological functions and clinical significance (Table 1). First, circRNAs are differentially expressed in almost all cancers, including lung cancer, gastrointestinal cancers and urological cancers. Second, circRNAs are differentially expressed, rather than simply upregulated or downregulated in the same tumor. For example, circPVT1 and circFADS2 are upregulated [32, 33] and circNOL10 and circPTPRA are downregulated in lung cancer [29, 34]. Third, the same circRNAs are also differentially expressed in a variety of tumors, but the upregulated or downregulated expression patterns are not the same in different tumors. CircHIPK3 is upregulated in most types of cancers, such as gastric cancer, colorectal cancer and hepatocellular carcinoma [35, 36, 39, 41]. However, it is downregulated in bladder cancer [43]. Based on these findings, the expression pattern of circRNAs in malignant tumors is very complex, and thus a high-throughput detection method is needed to determine the differential expression profile of circRNAs to further understand the importance of the differential expression patterns of circRNAs in cancer.

**Table 1:** Representative circRNAs with clear mechanisms, biological functions and clinical significance in cancers

Mechanism	Cancer	CircRNA	Expression	Biological functions	Clinical significance	Ref
Transcriptional regulation	Lung cancer	circNOL10	Down	Inhibit cell proliferation and promote cell apoptosis;	Lung cancer differentiation	[29]
	Colorectal Cancer	circITGA7	Down	Inhibit cell growth and metastasis;	Tumor size; Lymph metastasis; Distant metastasis and TNM stage;	[30]
	Breast Cancer	circFECR1	Up	Promotes tumor metastasis;	Metastases; Advanced stages;	[31]
MiRNA sponging	Lung cancer	circPVT1	Up	Promote cell proliferation and Inhibit cell apoptosis;	Tumor size; TNM stage; Overall survival;	[32]
		circFADS2	Up	Promote proliferation and invasion;	TNM; LNM; Overall survival;	[33]
		circPTPRA	Down	Inhibit proliferation and migration;	Metastasis; Overall survival;	[34]
	Gastric Cancer	circHIPK3	Up	Promote proliferation and migration;	Overall survival; Infiltrative type GC cell; Advanced TNM stage;	[35, 36]
		circPVT1	Up	Promote proliferation;	Overall survival; Disease-free survival;	[37]
		circLARP4	Down	Inhibit proliferation and invasion;	Overall survival;	[38]
	Colorectal cancer	circHIPK3	Up	Promote proliferation, migration, invasion, metastasis, autophagy and inhibit apoptosis;	Advanced TNM stage; Lymph node metastasis, Distant metastasis; Advanced tumor;	[39]
		circ_0021977	Down	Inhibit proliferation, migration, and invasion;	TNM stage; Overall survival;	[40]
	Hepatocellular carcinoma	circHIPK3	Up	Proliferation, migration	Tumor differentiation; Advanced TNM stage; HBV-DNA copy numbers; Liver cirrhosis;	[41]
		circ_0001649	Down	Inhibit proliferation and migration	Differentiation and tumor Satellite;	[42]
	Bladder cancer	circHIPK3	Down	Inhibit Migration, invasion and angiogenesis.	Advanced tumor; Lymph node metastasis;	[43]
Protein interaction	Gastric cancer	circ-DONSON	Up	Facilitate cancer growth and invasion	TNM stage; Lymph node metastasis; Overall survival and Disease-free survival;	[44]
		circAGO2	Up	Promote growth, invasion, and metastasis	Metastasis; Overall survival;	[45]
Self-translation	Glioblastoma	circ-SHPRH	Down	Suppress tumor progression and tumorigenesis	Overall survival;	[46]
		circFBXW7	Down	Inhibit proliferation and cell cycle acceleration.	Overall survival;	[47]

*Ref* Reference

The complex expression pattern of circRNAs also implies complex biological functions and clinical significance in cancer. CircRNAs modulate multiple biological functions of cancer, such as proliferation, metastasis and angiogenesis [30, 31]. For example, circPVT1 promotes cell proliferation and inhibits cell apoptosis in lung cancer [32] and gastric cancer [37], while circHIPK3 inhibits angiogenesis in bladder cancer [43]. CircRNAs have important tumor-related clinical significance and are associated with various clinical features of cancer and patient outcomes. As shown in Table 1, the clinical features of cancer mainly include cancer differentiation, lymph node metastasis, distant metastasis and the TNM stage [40, 42]. The association between circRNAs and the clinical characteristics of tumors suggests the potential diagnostic value of circRNAs. For example, low circNOL10 expression may indicate a low level of differentiation of lung cancer [29]. High circ-DONSON expression suggests that clinicians should closely monitor lymph node metastasis in patients with gastric cancer [44]. The main circRNA-related indicators of outcomes in patients with cancer are overall survival and disease-free survival [44, 45]. In patients with gastric cancer, high circPVT1 expression indicates poor overall survival and disease-free survival [37], while high circLARP4 expression is related to longer overall survival [38]. CircFBXW7 and circ-SHPRH are good prognostic biomarkers for glioblastoma because their high expression results in longer overall survival [46, 47]. In conclusion, the important role of circRNAs in tumors must not be ignored. Moreover, the crucial role of circRNAs in mediating anticancer drug resistance is emerging [48]. Here, we are the first to summarize the role of circRNAs in anticancer drug resistance from the perspective of drug classification.

## Nonplatinum cytotoxic drugs

Traditional anticancer drugs can be divided into cell cycle-specific anticancer drugs and cell cycle-nonspecific anticancer drugs according to their cellular kinetics [49, 50]. Classical cell cycle-specific drugs include topoisomerase inhibitors, antimetabolite drugs and some drugs derived from plants [51–53]. Typical cell cycle-nonspecific anticancer drugs are anticancer antibiotics [54].

### Topoisomerase inhibitors

Topoisomerase is abundantly distributed in all eukaryotic nuclei and resolves topological problems during bioprocesses such as DNA replication and DNA transcription [55]. In the 1970s, camptothecin was found to have anticancer activity, and topoisomerase I was identified as its major target [56]. Irinotecan is an S-phase-specific camptothecin derivative that exerts efficient anticancer activity against metastatic colorectal cancer (CRC) [57]. However, clinical resistance to irinotecan is common [58]. Jian et al. [59] found that circ_001680 increases migration and is involved in irinotecan resistance in CRC. Circ_001680 acts as an oncogene by sponging miR-340 to upregulate BMI1 in CRC. BMI1 has been recognized as a positive regulator that induces cancer stem cell-like properties [60, 61], which are deeply involved in irinotecan resistance [62, 63]. Sphere formation assays showed that SW480 and HCT116 cells with circ_001680 overexpression formed more stem cell spheres after treatment with irinotecan and had a more robust cell growth ability than control cells. In vivo, the sizes and weights of tumors in the control group were markedly decreased after treatment with irinotecan but were not significantly changed in the circ_001680-overexpressing group. These results indicate that the circ_001680/miR-340/BMI1 axis may contribute to irinotecan resistance by regulating cancer stem cell-like properties.

### Antimetabolite drugs

Pemetrexed is a folate antagonist that disrupts folate-dependent metabolic processes [64, 65]. It functions mainly during S-phase. Xu et al. [66] reported a novel circRNA, circMTHFD2, related to pemetrexed resistance in gastric cancer. Overexpression of circMTHFD2 was confirmed in pemetrexed-resistant gastric cancer cells. Further investigation showed that circMTHFD2 could bind with miR-124 and promote the protein expression of FDZ5 and MDR-1 to induce pemetrexed resistance. 5-Fluorouracil (5-FU), a fluoropyrimidine, can inhibit thymidylate synthase to induce thymineless cell death and functions mainly as an S-phase antimetabolite [67, 68]. 5-FU has been the standard treatment for many solid tumors, including CRC, for decades [69, 70]. The benefit of 5-FU is always limited by the development of resistance, and the specific molecular mechanisms are complex [71]. Xiong et al. [72] identified 71 differentially expressed circRNAs in 5-FU-resistant CRC cells by microarray hybridization. Forty-seven of these circRNAs were upregulated, and 24 were downregulated. These circRNAs were distributed on each chromosome, but most were located on chromosomes 1, 8, and 9 (11, 10, and 10%, respectively), suggesting that these 3 chromosomes are more strongly correlated with 5-FU resistance than other chromosomes. The top 3 most strongly upregulated circRNAs were predicted to be capable of regulating the Wnt signaling pathway, which is deeply involved in 5-FU resistance [73]. In addition, several 5-FU-resistance-related miRNAs, such as miR-885-3p [74], may be targets of these circRNAs.

Gemcitabine is another widely used S-phase antimetabolite drug [75]. Yan et al. [76] reported that circ_0035483 is significantly increased in TK10 and UO31 renal cancer cell lines after treatment with gemcitabine. The results of an MTT assay showed that high expression of circ_0035483 in gemcitabine-treated TK10 cells could enhance cell viability to induce gemcitabine resistance. The results of RNA pulldown experiments revealed that circ_0035483 can bind with miR-335 as a sponge. Further dual-luciferase assay results identified CCNB1 as a target of miR-335. CCNB1 is an important checkpoint molecule in the cell cycle and is deeply involved in cell cycle-specific gemcitabine resistance [77, 78]. Promotion of circ_0035483 expression increased the expression of CCNB1, and cell apoptosis was inhibited. Thus, circ_0035483 promotes gemcitabine resistance in human renal cancer cells by sponging miR-335 to upregulate CCNB1.

Gemcitabine resistance can also be developed by selection under exposure to an increasing gradient of gemcitabine in pancreatic cancer (PC) cell lines [79]. The gemcitabine-resistant SWl990/GZ cell line was developed from the parental SWl990 cell line, and 81 circRNAs were found to be differentially expressed between this cell line and the parental cell line [80]. Twenty-six of these circRNAs were upregulated, and 55 were downregulated. Four circRNAs were validated by qRT-PCR. Pathway analysis indicated that these dysregulated circRNAs are deeply involved in gemcitabine resistance in PC via modulation of MAPK and mTOR signaling pathways [81, 82].

In addition, Shao et al. [83] established the gemcitabine-resistant PANC-1-GR cell line and compared the differential circRNA profiles between the PANC-1-GR cell line and the parental PANC-1 cell line. A total of 126 circRNAs were identified by high-throughput transcriptome sequencing as significantly differentially expressed in these two cell lines; 68 of which were upregulated and 58 downregulated. Moreover, gene ontology (GO) term and pathway analysis results indicated the functions of these circRNAs in PC progression-related signaling pathways, such as the ErbB pathway [84]. The two most significantly upregulated circRNAs (chr14:101402109–101,464,448+ and chr4:52729603–52,780,244+) were further validated, and functional experiments showed that silencing these two circRNAs restored gemcitabine sensitivity in PANC-1-GR cells, while overexpression of these circRNAs promoted gemcitabine resistance in both the PANC-1 and MIA PACA-2 cell lines. Identification of potential binding miRNAs of these two circRNAs by sequence analysis suggested that miR-145-5p, a tumor suppressor inhibiting PC progression [85], could bind both circRNAs and act as a downstream target. Moreover, the plasma expression levels of the two circRNAs and miR-145-5p in gemcitabine-treated PC patients were verified via qRT-PCR. Consistent with previous results, the two circRNAs were upregulated but miR-145-5p was downregulated in the plasma of gemcitabine-resistant patients. This finding confirms their potential for monitoring gemcitabine resistance via liquid biopsy.

### Drugs derived from plants

Taxol is an M-phase specific plant drug that was originally derived from the bark of the Pacific yew and has become one of the most widely used agents [86]. Unlike antimetabolite drugs, Taxol can bind tubulin to arrest mitosis and induce apoptosis, resulting in cell death [87]. The mechanism underlying Taxol resistance remains poorly understood [88]; however, ncRNA dysregulation plays an important role in this process [89, 90]. Taxol is an effective chemotherapeutic drug in first-line therapy for gastric cancer [91]. Liu et al. [92] reported that circPVT1, a circRNA derived from the oncogenic lncRNA PVT1 locus [93], can mediate Taxol resistance in gastric cancer. Overexpression of circPVT1 was confirmed both in Taxol-resistant gastric cancer tissues and cells, and circPVT1 knockdown promoted apoptosis triggered by Taxol. ZEB1 is a well-known promoter of Taxol resistance [94, 95]. Mechanistically, circPVT1 can promote ZEB1 expression by sponging miR-124-3p. In addition, Taxol resistance remains problematic in the treatment of ovarian cancer [96, 97]. Expression profiles of dysregulated circRNAs were identified in Taxol-resistant ovarian cancer tissues. A total of 833 aberrantly expressed circRNAs were found; 341 were upregulated and 492 downregulated [98]. Among these circRNAs, circ_0063809 was further confirmed by qPCR to be upregulated in both Taxol-resistant tissues and cells. In vitro and vivo experiments demonstrated that silencing circ_0063809 promotes Taxol-induced cytotoxicity in Taxol-resistant ovarian cancer cells. Moreover, the results of mechanistic experiments revealed that circ_0063809 can upregulate FOXR2 expression by sponging miR-1252 to contribute to Taxol resistance in ovarian cancer. Taxol is also recommended for first-line treatment of HER2-negative metastatic breast cancer (BC) [99]. CircAMOTL1 was found to contribute to Taxol resistance by activating the AKT pathway [100].

Xu et al. [101] reported the differentially expressed circRNA profile in the Taxol-resistant A549/Taxol cell line compared to the parental A549 cell line. A total of 11,281 circRNAs were identified, among which 2909 circRNAs were upregulated and 8372 were downregulated. GO analysis results showed that the most significantly enriched terms were linked to Taxol resistance-related terms, such as GTPase binding and alterations in cell adhesion [102, 103]. In addition, Cytoscape was used to visualize the circRNA-miRNA interaction network. Several miRNAs previously reported to drive Taxol resistance were predicted. For example, miR-141 has been suggested to induce Taxol resistance in ovarian cancer [104], and miR-34c-5p can downregulate p53 to induce Taxol resistance in lung cancer [105]. Circ_0071799, which was upregulated, is related to miR-141. Downregulated circ_0091931 can interact with miR-34c-5p. In addition, Li et al. [106] reported that circ_0002483 overexpression can enhance Taxol sensitivity in non-small cell lung cancer (NSCLC). Circ_0002483 has been confirmed by a dual-luciferase reporter assay to sponge miR-182-5p, and pathway analysis revealed that the circ_0002483/miR-182-5p interaction may be deeply involved in the FoxO signaling pathway. Further analysis indicated that the 3′ untranslated regions (3′-UTRs) of GRB2, FOXO1 and FOXO2 contain miR-182-5p complementary sequences. Mechanistically, the circ_0002483/miR-182-5p/GRB2/FOXO1/FOXO3 axis can reverse Taxol resistance in NSCLC.

Yu et al. [107] evaluated the overexpression of circ_0003998 in the docetaxel-resistant lung adenocarcinoma A549/DTX and H1299/DTX cell lines. Knockdown of circ_0003998 in A549/DTX and H1299/DTX cells partially restored docetaxel sensitivity via promotion of cell apoptosis. In addition, miR-326 was predicted by the Circular RNA Interactome (CircInteractome) database [108] as a potential target of circ_0003998. MiR-326 is a well-recognized tumor suppressor, and its downregulation is deeply involved in multidrug resistance in tumors [109], and a significant negative correlation between circ_0003998 expression and miR-326 expression was found in lung adenocarcinoma tissues. Further dual-luciferase reporter assays confirmed the direct binding between circ_0003998 and miR-326. In addition, miR-326 inhibitors were found to significantly reverse the increased sensitivity to docetaxel caused by circ_0003998 siRNA. In summary, overexpression of circ_0003998 induces docetaxel resistance in lung adenocarcinoma partially by sponging of miR-326. Docetaxel can also be used in first-line chemotherapy for patients with metastatic nasopharyngeal carcinoma (NPC) [110]. Hong et al. [111] reported that circCRIM1 binds to miR-422a to upregulate FOXQ1 in NPC. FOXQ1 is intimately involved in chemotherapeutic resistance by regulating the epithelial-mesenchymal transition (EMT) [112]. In vivo and in vitro functional experiments have confirmed that FOXQ1 overexpression induced by circCRIM1 also contributes to metastasis and docetaxel resistance in NPC [111].

### Anticancer antibiotics

As a cell cycle-nonspecific anticancer drug, doxorubicin (Adriamycin) is an anthracycline antibiotic antineoplastic drug widely used in the clinic [113]. Its underlying anticancer mechanisms are inhibition of DNA synthesis, interference with topoisomerase II activity and induction of free radical damage to cells [54]. CircRNAs have also been reported to play a role in doxorubicin resistance. The differential expression profile of circRNAs in a doxorubicin-resistant acute myeloid leukemia (AML) cell line (THP-1/ADM) was identified by analysis of a human circRNA array. Forty-nine circRNAs were found to be differentially expressed with a fold change of > 2 in THP-1/ADM cells compared to THP-1 cells. Thirty-five were upregulated, and 14 were downregulated. Among these circRNAs, circPAN3 was considered a candidate mediator of doxorubicin resistance due to its substantial differential expression and the important role of its host gene PAN3 in AML [114]. In addition, circPAN3 expression was higher in the bone marrow of patients with refractory/recurrent AML than in the bone marrow of doxorubicin-sensitive patients. Moreover, knockdown of circPAN3 reversed doxorubicin resistance in THP-1/ADM cells. TargetScan showed that miR-153-5p and miR-183-5p could be target genes of circPAN3. XIAP, a well-known chemoresistance-related gene in AML [115], was found to be a downstream target of both miR-153-5p and miR-183-5p. Further mechanistic experiments also confirmed the circPAN3/miR-153-5p/miR-183-5p/XIAP axis, which contributes to doxorubicin resistance in AML.

Doxorubicin has also been approved by the FDA for clinical use in patients with BC [116]. The results of a microarray screen also indicated the important role of circRNAs in doxorubicin resistance in BC [117]. Eighteen circRNAs were identified as significantly differentially expressed between doxorubicin-resistant MCF-7/ADM and doxorubicin-sensitive MCF-7 cells; 12 were upregulated, while 6 were downregulated. Kyoto Encyclopedia of Genes and Genomes (KEGG) analysis revealed that the most relevant signaling pathways included the MAPK and PI3K/Akt signaling pathways, which are closely related to doxorubicin resistance [118, 119]. In addition, circ_0006528 expression was measured by qRT-PCR. The results confirmed circ_0006528 overexpression in doxorubicin-resistant cell lines and tissues. Downregulation of circ_0006528 restored the sensitivity of MCF-7/ADM and MDA-MB-231/ADM cells to doxorubicin. Moreover, miR-7-5p, which has been reported to be involved in PI3K/Akt signaling pathway activation and chemoresistance in BC [120], was predicted to be a target of circ_0006528, while Raf1, a MAPK signaling pathway activator, is a direct target of miR-7-5p [121]. Further experimental results proved that the circ_0006528/miR-7-5p/Raf1 axis may be responsible for doxorubicin resistance in BC.

CircKDM4C was reported by Liang et al. to be a tumor suppressor in BC [122]. In vitro experiments identified decreased expression of circKDM4C in MDA-MB-231/DOX cells compared with the parental MDA-MB-231 cells. CircKDM4C knockdown in MDA-MB-231 cells promoted resistance to doxorubicin, while overexpression of circKDM4C in MDA-MB-231/DOX cells inhibited cell proliferation, enhanced apoptosis and finally reversed doxorubicin resistance in dose-dependent and time-dependent manners. MiR-548p was predicted by CircInteractome database analysis to be a target of circKDM4C, and upregulation of miR-548p was found to partially abolish circKDM4C overexpression-induced inhibition of doxorubicin resistance. Further miRNA target prediction indicated that the 3′-UTR of phenazine biosynthesis-like domain-containing protein (PBLD) is considered a putative target of miR-548p. PBLD is associated with various tumor progression-related signaling pathways, such as the MAPK pathway [123]. Luciferase reporter assay results confirmed the circKDM4C/miR-548p/PBLD axis, and the contribution of this axis to doxorubicin resistance was proven both in vitro and in vivo.

## Platinum drugs

Platinum drugs such as cisplatin and oxaliplatin are widely used in the treatment of human cancers and have achieved clinical success as standard therapies [124]. Oxaliplatin is a platinum complex with oxalate and (1R,2R)-1,2-diaminocyclohexane (DACH) ligands. Oxaliplatin uptake has been reported to be mediated by the organic cation transporters OCT1 and OCT2, which are overexpressed in CRC cells [125]. This overexpression partially explains why oxaliplatin is an efficient component in the adjuvant FOLFOX treatment regimen in metastatic CRC. Oxaliplatin resistance has become an issue in CRC. Drug-resistant HCT116 (HCT116-R) cells were developed by exposure to FOLFOX, and circRNA microarray analysis was performed to compare circRNA expression profiles between HCT116-R and parental HCT116 cells [126]. A total of 773 upregulated and 732 downregulated circRNAs were identified. The presence of two upregulated circRNAs, circ_32883 and circ_0338, in extracellular vesicles was further validated. Circ_32883 was found to be significantly upregulated in CRC tissues. Several microRNAs, such as miR-501-5p, were predicted to interact with circ_32883 to bind to their target genes. Hon et al. [127] isolated exosomes from the cell culture medium of HCT116-R and parental HCT116 cells and used by circRNA microarray analysis to identify 105 significantly upregulated and 34 downregulated circRNAs. Consistent with a previous report, circ_32883 and circ_0338 were upregulated in HCT116-R cell-derived exosomes. In addition, the resistance of HCT116-R cells to FOLFOX was partially reversed by knockdown of circ_0338, indicating a potential role for circ_0338 as a biomarker for FOLFOX resistance in CRC. Wang et al. [128] identified a novel circRNA, circ_0005963, in exosomes derived from oxaliplatin-resistant CRC cells. This circRNA could be transferred into oxaliplatin-sensitive CRC cells and promote glycolysis and oxaliplatin resistance in the recipient cells. Further experiments showed that circ_0005963 can sponge miR-122 to upregulate PKM2. In addition, si-circ_0005963 transferred by exosomes inhibited glycolysis and reversed resistance to oxaliplatin. Thus, this exosomally initiated circ_0005963/miR-122/PKM2 signaling axis has great therapeutic potential for oxaliplatin-resistant CRC.

Cisplatin was first synthesized by Michele Peyrone and was approved by the US FDA for use in testicular and ovarian cancer in 1979 [129]. Cisplatin often leads to an initial therapeutic response, but many originally sensitive tumors gradually develop resistance [130]. Huang et al. [131] screened 31 circRNAs derived from EIF3a, the largest subunit of the translation initiation factor EIF3, in cisplatin-resistant A549/DDP cells. Two circRNAs transcribed from EIF3a (circ_0004350 and circ_0092857) were validated to exhibit differential expression between A549/DDP and parental A549 cells. Moreover, downregulation of circ_0004350 and circ_0092857 reversed cisplatin resistance in lung cancer cells. Functional enrichment analysis revealed that these two circEIF3as may have a synergistic functional effect with their parental EIF3a gene on cisplatin resistance.

Circ_0076305 is another circRNA identified to promote cisplatin resistance in lung cancer. Significantly elevated expression of circ_0076305 was proven in cisplatin-resistant tissues and cells [132]. Moreover, cisplatin treatment was found to effectively upregulate the expression of circ_0076305 in A549 and H1650 cells. Further gain- and loss-of-function experiments indicated that circ_0076305 can regulate cisplatin resistance in lung cancer cells. Bioinformatic and mechanistic experiments such as circRNA immunoprecipitation (circRIP) and luciferase reporter assays were used to validate the binding of circ_0076305 to miR-296-5p as well as to miR-296-5p and STAT3. Reports have indicated that miR-296-5p acts as a tumor suppressor in lung cancer and that STAT3 can contribute to cisplatin resistance [133, 134]. Pearson correlation analysis showed that the expression levels of circ_0076305 are negatively correlated with those of miR-296-5p and positively correlated with those of STAT3. These results suggest that circ_0076305 increases cisplatin resistance by acting as a miR-296-5p sponge to promote STAT3 expression in lung cancer. Zheng et al. [135] reported that circPVT1 contributes to cisplatin resistance in lung adenocarcinoma via the miR-145-5p/ABCC1 axis. However, circRNAs can also act as suppressors of cisplatin resistance in lung cancer. Circ_0001946 expression was significantly lower in A549/DDP cells than in parental A549 cells [136]. The 1485 nt long circ_0001946 is derived from cerebellar degeneration-related protein 1 (CDR1). Knockdown of this circRNA was found to increase cisplatin resistance in A549 cells, and the results of a host cell reactivation (HCR) assay and Western blot analysis demonstrated that silencing circ_0001946 activates the nucleotide excision repair (NER) signaling pathway, which increases the ability for DNA damage repair [137], to reduce cisplatin sensitivity in lung cancer.

Treatments for osteosarcoma (OS), the most common malignant bone tumor in teenagers, are limited [138]. Cisplatin-based neoadjuvant chemotherapy has greatly improved the 5-year survival rate of OS to 70–80%, but patients with resistance to cisplatin still have poor clinical outcomes [139]. Zhu et al. [140] reported dysregulation of eighty circRNAs in paired cisplatin-based chemotherapy-resistant and cisplatin-based chemotherapy-sensitive OS cell lines, and bioinformatic analysis indicated that these circRNAs are significantly related to drug metabolism. In addition, the most strongly upregulated circRNA, circ_0004674, was confirmed in both tissues and cells. Patients with high circ_0004674 expression have shorter overall survival times than those with low circ_0004674 expression. These results indicate a potential crucial role of circRNAs in cisplatin-based chemotherapeutic resistance in OS. Moreover, Zhu et al. constructed competing endogenous RNA (ceRNA) networks on the basis of bioinformatic analysis combined with well-known drug resistance-related genes and signaling pathways. The circ_0001258/miR-744-3p/GSTM2 axis was confirmed by mechanistic studies to contribute to resistance to cisplatin-based chemotherapeutics in OS [141]. Zhang et al. [142] found that circ_001569 can promote cisplatin resistance by activating the Wnt/β-catenin signaling pathway in OS cells. Overexpression of circPVT1 in chemo resistant OS patients has also been reported, and this pattern indicated poor prognosis [143]. In addition, serum circPVT1 expression was found to be increased in OS patients compared with healthy controls, indicating the possible clinical significance of serum circPVT1 as a biomarker in OS. The results of PCR and Western blot assays confirmed that CircPVT1 knockdown reduces the expression of ABCB1, a classical multidrug resistance-related gene [144], in cisplatin-resistant OS cells. In addition, the serum circ_0081001 level was found to be increased in OS patients and to gradually increase during treatment in OS patients who ultimately developed resistance to cisplatin-based chemotherapeutics. Dynamic monitoring of serum circ_0081001 may promptly and accurately reflect the changes in the chemo response of OS patients [145]. These results demonstrate the important role of circRNAs in resistance to cisplatin-based chemotherapeutics in OS. Serum circRNAs could be therapeutic biomarkers in OS.

Cisplatin-based chemotherapeutic regimens are also widely used for the treatment of other solid tumors, including hepatocellular cancer (HCC) [146], gastric cancer [147], bladder cancer [148], ovarian cancer [149] and thyroid cancer [150]. Luo et al. [151] identified circRNA_101505 as an inhibitor of cisplatin resistance in HCC. Low expression of circRNA_101505 was found to be correlated with cisplatin resistance and to indicate poor prognosis in HCC. Overexpression of circRNA_101505 sensitizes resistant HCC cells to cisplatin both in vitro and in vivo. NOR1 is a tumor suppressor that can inhibit cisplatin-induced autophagy, leading to increased cisplatin cytotoxicity and apoptosis in cancer [152]. Mechanistic studies demonstrated that upregulation of circRNA_101505 can promote NOR1 expression by sponging miR-103, thus reversing cisplatin resistance in HCC. In addition, both circ_0081143 and circAKT3 are upregulated in cisplatin-resistant gastric cancer. Circ_0081143 promotes CDK6 activity by sponging miR-646, and circAKT3 acts as an endogenous sponge by directly binding miR-198 to upregulate PIK3R1, resulting in enhanced cisplatin resistance in gastric cancer [153, 154]. CircFN1 has also been reported to enhance cisplatin resistance by sponging miR-182-5p in gastric cancer [155]. Analysis of circRNA microarray data for cisplatin-resistant ovarian cancer tissues revealed that 339 circRNAs were aberrantly expressed with a 2-fold change [156]. Among these circRNAs was the circRNA Cdr1as, whose downregulated expression in cisplatin-resistant tissues and cells was further confirmed by qPCR. Overexpression of Cdr1as reversed the cisplatin resistance of ovarian cancer cells both in vitro and in vivo. Mechanistic studies proved that Cdr1as can function as a molecular sponge of miR-1270 to upregulate SCAI, which can promote cisplatin sensitivity in ovarian cancer. Chi et al. [157] reported that the serum circ_0000285 level was decreased in cisplatin-resistant bladder cancer patients compared to cisplatin-sensitive bladder cancer patients and that a low circ_0000285 level indicated poor prognosis, suggesting circRNA_000285 as a biomarker for the cisplatin treatment response in bladder cancer. In addition, Chen et al. [158] found that deletion of circFNTA reduces cisplatin resistance in bladder cancer. High expression of circFNTA can activate KRAS signaling via the miR-370-3p/FNTA axis, thereby contributing to cisplatin resistance.

## Endocrine drugs

Endocrine therapy is the mainstay option for patients with hormone-sensitive breast and prostate cancer [159]. The androgen receptor (AR) plays a key role in the tumorigenesis of prostate cancer and is thus an effective therapeutic target in prostate cancer [160, 161]. Antiandrogenic drugs such as flutamide and enzalutamide can control the progression of prostate cancer via maximal androgen blockade [162, 163]. However, the development of castration resistance is inevitable. Cao et al. [164] identified 13 circRNAs derived from AR and found overexpression of these circRNAs during progression of prostate cancer to castration-resistant prostate cancer. Moreover, these circRNAs were detectable in serum samples from castration-resistant prostate cancer patients, indicating their potential roles as biomarkers. A circRNA microarray was used to screen differentially expressed circRNA profiles in enzalutamide-resistant prostate cancer cells [165]. A total of 278 circRNAs were significantly upregulated, and 588 were downregulated. Among the downregulated circRNAs, downregulation of circ_0004870 was confirmed. Downregulated expression of this circRNA may promote the expression of AR-V7 via U2AF65 to contribute to enzalutamide resistance. Wu et al. [166] reported that circRNA17 suppression can lead to an increase in the AR-V7 level, thus leading to enzalutamide resistance. Instead of acting as a sponge, circRNA17 upregulates miR-181c-5p by maintaining its stability. MiR-181c-5p can bind the 3′-UTR of AR-V7 to decrease its expression. Functional experiments further proved that the circRNA17/miR-181c-5p/AR-v7 signaling axis alters enzalutamide resistance. CircUCK2 was also found to be downregulated in enzalutamide-resistant prostate cancer cells, indicating its role as a suppressor of enzalutamide resistance [167]. Upregulated circUCK2 can sponge miR-767-5p to upregulate TET1.

Tamoxifen (TAM) remains a cornerstone in the treatment of BC patients with estrogen receptor-positive tumors and has significantly improved the clinical outcome of BC patients over the past decades [168, 169]. Resistance to TAM limits its clinical benefit. The mechanisms underlying TAM resistance are complex, and ncRNAs such as circRNAs play an important role in this process [170, 171]. Sang et al. [172] used a sequencing approach to compare the circRNA profile of the TAM-resistant BC cell line MCF7/TR to that of its parental cell line MCF7. A total of 352 circRNAs were significantly upregulated, and 113 were downregulated. Among the downregulated circRNAs in MCF7/TR cells was circ_0025202, which was negatively correlated with tumor progression in HR-positive BC. The results of in vitro experiments showed that circ_0025202 expression can inhibit the malignant phenotype and TAM resistance of MCF7/TR cells, and the results of the IC50 assay demonstrated that low circ_0025202 expression contributes to TAM resistance. Bioinformatic analysis indicated that circ_0025202 may act as a miRNA sponge. Further experiments proved that circ_0025202 can upregulate FOXO3a, an inhibitor of TAM resistance in BC [173], by sponging miR-182-5p, leading to reversion of TAM resistance. Liang et al. [174] identified a novel circRNA, circBMPR2, as a suppressor of TAM resistance in BC. Upregulated circBMPR2 can sponge miR-553 to prevent it from inhibiting the tumor suppressor USP4 [175] and mitigate TAM resistance. Thus, the circBMPR2/miR-553/USP4 axis may be a therapeutic target in TAM-resistant BC.

## Targeted and immunotherapeutic drugs

Targeted therapy has been widely used in the clinic due to its excellent efficacy [176]. Small molecule inhibitors that target the epidermal growth factor receptor (EGFR) tyrosine kinase, such as gefitinib and osimertinib (AZD9291), have ushered in a new era in the treatment of NSCLC patients with activating EGFR mutations [177]. However, acquired resistance to EGFR tyrosine kinase inhibitors (EGFR-TKIs) remains inevitable [178]. Liu et al. [179] reported that circRNAs may act as predictive biomarkers for the efficacy of gefitinib therapy. A total of 1377 differentially expressed circRNAs in the plasma of patients with EGFR-mutant NSCLC were identified by microarray analysis; of these circRNAs, 989 were upregulated and 388 were downregulated. RT-qPCR was used to further validate the differential expression of circ_0109320 and circ_0134501 in an independent cohort of gefitinib-treated NSCLC patients. Pearson correlation analysis indicated a positive correlation between circ_0109320 expression and progression-free survival (PFS), and the Kaplan-Meier survival curve showed that patients with high circ_0109320 expression had longer PFS times. In addition, Zhou et al. [180] reported a novel circRNA, circ_0004015, involved in gefitinib resistance. Circ_0004015 overexpression was found to contribute to the development of gefitinib resistance in HCC827 cells. Mechanistically, circ_0004015 sponges miR-1183. PDPK1 is a classic effector of the epidermal growth factor (EGF) signaling pathway that can prevent apoptosis and mediate drug resistance in cancer [181, 182]. PDPK1 was proven to be a target gene of miR-1183. Functional assays demonstrated that circ_0004015 induces gefitinib resistance via regulation of the miR-1183/PDPK1 axis. Osimertinib is a third-generation EGFR-TKI that can overcome the resistance to first-generation EGFR-TKIs caused by the T790M mutation [183, 184]. However, acquired resistance to osimertinib is unavoidable and lacks effective countermeasures [185]. Chen et al. [186] successfully established osimertinib-resistant NSCLC cell lines (H1975/AZDR and HCC827/AZDR) and screened these cell lines for dysregulated circRNAs. A total of 15,504 differentially expressed circRNAs were identified, of which 7966 were upregulated and 7538 were downregulated. GO and KEGG analyses of circRNA host genes indicated their involvement in molecular functions such as DNA replication and signaling pathways such as the p53 signaling pathway, which directly correlate with cancer pathogenesis.

Fusion between BCR (chromosome 22q11.2) and ABL1 (chromosome 9q34) leads to chronic myeloid leukemia (CML) [187]. The first human malignancy to be treated with targeted drugs, CML has an 85% survival rate with imatinib therapy [188]. However, the emergence of resistance to imatinib has become a significant problem [189]. Pan et al. [190] identified a novel circRNA, circBA9.3, derived from BCR-ABL1 in CML patients. CircBA9.3 was found to be significantly overexpressed in imatinib-resistant CML patients. Upregulation of circBA9.3 contributes to imatinib resistance by promoting the proliferation and inhibiting the apoptosis of cells. Western blot analysis results demonstrated that circBA9.3 may upregulate ABL1 and BCRABL1 protein expression to induce imatinib resistance. Ping et al. [191] found that circ_100053 is significantly upregulated in the serum of CML patients with imatinib resistance, indicating that circ_100053 may be a biomarker for imatinib resistance in CML.

Currently, immunotherapy, represented by anti-PD-1/PD-L1 antibodies, has shown great efficacy in the clinical treatment of cancers such as melanoma [192]. The binding of PD-1 and PD-L1 can inhibit T cell activation and lead to tumor immune escape [193]. Therefore, blocking this signaling pathway with specific inhibitors can promote T cell recognition and killing of tumor cells [194]. Anti-PD-1/PD-L1 antibodies such as nivolumab, pembrolizumab, atezolizumab and durvalumab have been approved by the FDA for NSCLC treatment [195–197]. However, resistance to PD1/PD-L1 blockade therapy remains a major issue during treatment [198]. A retrospective analysis by Zhang et al. [199] of 20 NSCLC patients receiving anti-PD-1 antibody immunotherapy indicated that high circFGFR1 expression may contribute to resistance to anti-PD-1 agents. Further investigation proved that circFGFR1 can sponge miR-381-3p to upregulate CXCR4, which has been widely reported as an immunosuppressive element in the tumor environment to induce the exclusion of T lymphocyte-like CD8+ T cells and mediate resistance to anti-PD-1 therapy [200, 201]. On the other hand, CXCR4 blockade can promote the efficacy of anti-PD-1 therapy via mobilization of CD8+ T cells [202]. In addition, a negative correlation between circFGFR1 and CXCR4 expression and CD8+ T cell frequency was discovered in 210 pairs of NSCLC and matched nontumor tissues [199].

## Future potential

Cancer has become the leading cause of death worldwide. Hanahan and Weinberg proposed six basic hallmarks of cancer in 2000 [203]. These hallmarks are (1) sustaining proliferative signaling, (2) evading growth suppressors, (3) enabling replicative immortality, (4) activating invasion and metastasis, (5) inducing angiogenesis, and (6) resisting cell death. Two emerging hallmarks, reprogramming of energy metabolism and evading immune destruction, were added in 2011 [204]. A genetic imbalance underlies these hallmarks of cancer. The main cause of drug resistance is also the genetic dysregulation of cancer hallmarks. As ncRNAs dysregulation occurs in virtually all human cancers, ncRNAs are also strongly related to each cancer hallmark and subsequently contribute to drug resistance. Linear lncRNAs and miRNAs are relatively well-studied ncRNAs [205, 206]. For example, the loss of miR-7 in colorectal cancer promotes EGFR expression, the core of classic proliferative EGFR signaling pathway. Tumor proliferation increases and resistance to cetuximab, a widely used EGFR inhibitor in clinical practice, is established [207]. The lncRNA HULC triggers autophagy, a multifaceted regulator of cell death [208], by stabilizing Sirt1 and leads to chemotherapy resistance in HCC [209]. Our group also focused on ncRNA-related drug resistance [210]. As shown in our previous study, miR-224 affects the G1/S transition of the cell cycle and apoptosis by modulating the p21 (WAF1/CIP1)/pRb pathway and the intrinsic mitochondrial death pathway, thereby promoting the resistance to cisplatin in lung adenocarcinoma [211]. Moreover, the lncRNA UCA1 epigenetically silences CDKN1A expression to inhibit cell apoptosis and contributes to gefitinib resistance [212]. Compared with linear lncRNAs, circRNAs related to anticancer drug resistance have been studied relatively less frequently. Few research summaries of this topic are currently available. We propose that a review of the existing literature describing the roles of circRNAs in anticancer drug resistance will provide the direction for future circRNA research.

CircRNAs were first identified as plant viroids, yeast mitochondrial RNAs, and hepatitis δ virus genomes. They were previously considered RNA splicing errors with no biological functions because of their generally low expression and absence of protein-coding ability [213]. With the development of gene sequencing and microarray techniques, the emerging role of circRNAs in physiological and pathological conditions has been revealed in recent studies [214]. For example, circCdr1as helps maintain brain function, and animals with circCdr1as knockout display excitatory synaptic transmission-related neuropsychiatric disorders [28].

**Fig. 2:**
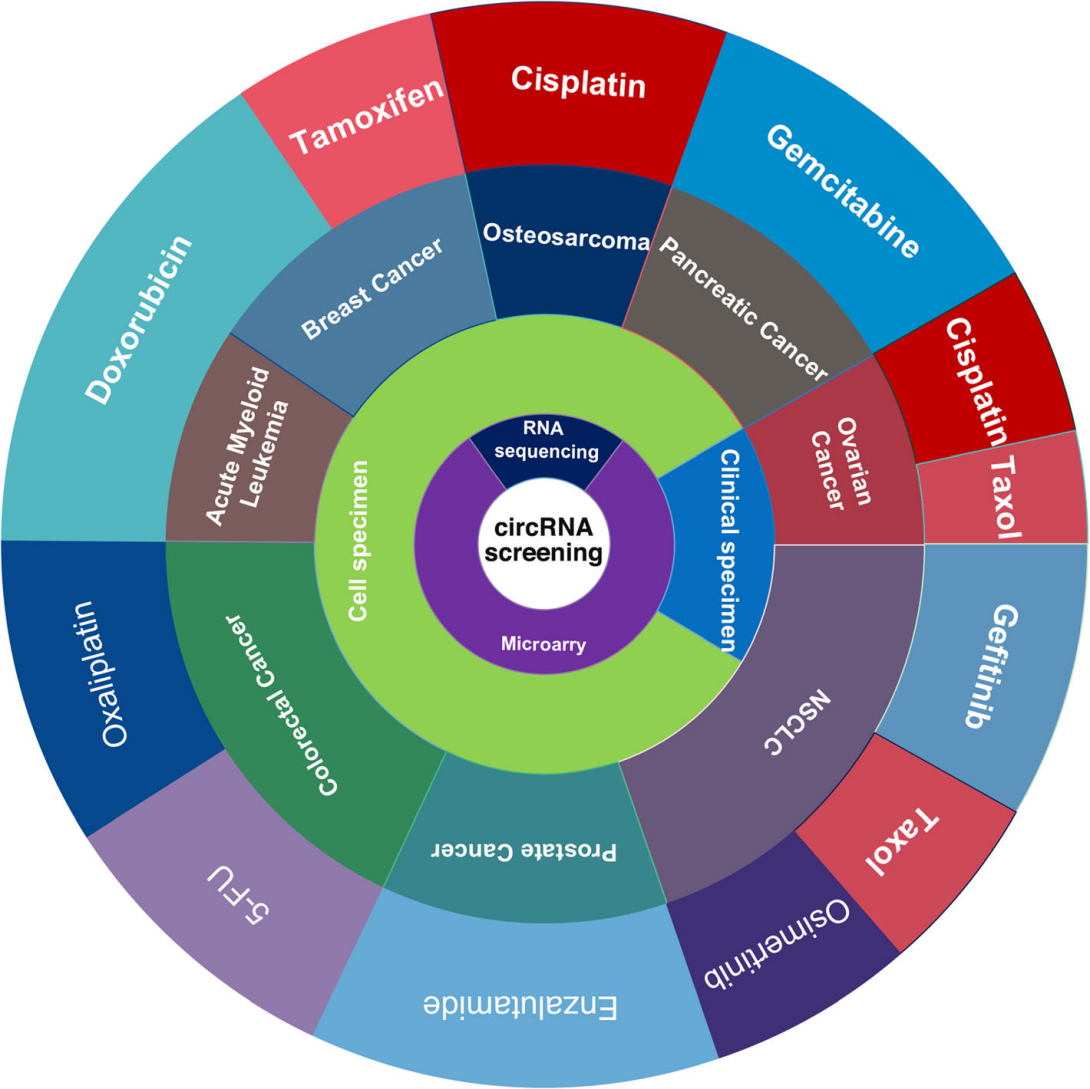
RNA sequencing and microarray analyses are effective methods to screen novel anticancer drug resistance-related circRNAs

At present, the main methods for detecting and quantifying circRNAs include RNA sequencing, microarray, PCR and northern blotting [26]. RNA sequencing and microarrays are characterized by high throughput, moderate accuracy and sensitivity [215, 216], while PCR is characterized by low throughput, high accuracy and sensitivity [217]. RNA sequencing also has the potential to identify novel circRNAs [215]. Northern blotting is the least expensive technique but has low throughput, accuracy and sensitivity, and thus it is relatively unsuitable for circRNAs research. As shown in Table 2 and Fig. 2, simultaneous profiling of large numbers of anticancer drug resistance-related circRNAs might be feasible using microarray and RNA sequencing techniques. PCR, on the other hand, is mainly used to validate the differential expression of a few key circRNAs after a bioinformatics analysis of data obtained from microarrays and RNA sequencing [217]. Therefore, RNA sequencing and microarrays are currently the best options for screening circRNAs related to anticancer drug resistance.

**Table 2:** Microarray and RNA sequencing of dysregulated circRNAs in anticancer drug resistance

Cancer	Drug classes	Drug	Specimen	Methods	DEGs		Bioinformation analysis	Ref
					UP	Down		
Prostate Cancer	Endocrine drugs	Enzalutamide	Enzalutamide resistance LNCaP cell models	Microarray	278	588	NA	[165]
Breast Cancer		Tamoxifen	MCF7/TR and MCF7/P cells	RNA sequencing	352	113	NA	[172]
	Anticancer antibiotics	Doxorubicin	MCF-7 and MCF-7/ADM cells	Microarray	12	6	MAPK and PI3K/Akt signaling pathway;	[117]
Acute Myeloid Leukemia			THP-1 and THP-1/ADM cells	Microarray	35	14	NA	[114]
Pancreatic Cancer	Antimetabolite drugs	Gemcitabine	SWl990 and SWl990/GZ cells	Microarray	26	55	MAPK and mTOR signaling pathways;	[80]
			PANC-1 and PANC-1-GR cells	RNA sequencing	68	58	ErbB pathway;	[83]
Colorectal Cancer		5-FU	HCT116 and CRR-HCT116 cells	Microarray	47	24	Wnt signaling pathway;	[72]
	Platinum drugs	Oxaliplatin	HCT116-P (parental) and HCT116-R cells	Microarray	773	732	Protein-binding activity;	[126]
			Exosomes from HCT116-P (parental) and HCT116-R cells	Microarray	105	34	Longevity regulating pathway; Wnt signaling pathway; cGMP-PKG signaling pathway;	[127]
Osteosarcoma		Cisplatin	Paired resistant and sensitive OS cell lines (MG63, KHOS and U2OS)	RNA sequencing	57	23	Glycosphingolipid biosynthesis–globo series; Linoleic acid metabolism;	[140]
Ovarian Cancer			Cisplatin-sensitive and cisplatin-resistant tissues	Microarray	148	191	NA	[156]
	Drugs derived from plants	Taxol	Taxol-sensitive and Taxol-resistant ovarian cancer tissues	Microarray	341	492	NA	[98]
NSCLC			A549 and A549/Taxol cells	Microarray	2909	8372	Small GTPase binding; Integrin signaling pathway;	[101]
	Targeted drug	Gefitinib	Plasma samples	Microarray	989	388	Biological processes of various cancers such as cancers of hematopoietic and lymphoid tissues, bladder cancer, colorectal cancer, and neuroblastoma;	[179]
		Osimertinib	H1975/AZDR and H1975; HCC827/AZDR and HCC827;	Microarray	7966	7538	P53, mTOR, and focal adhesion signaling pathways;	[186]

*Ref* Reference

CircRNA-induced drug resistance is not limited to traditional chemotherapeutic drugs, advanced targeted drugs and immunotherapeutic drugs are also affected (Tables 3, 4 and 5). Moreover, circRNA-related drug resistance affects a wide range of cancer types. These observations demonstrate the ubiquity of circRNAs in mediating anticancer drug resistance. In addition, although most circRNAs mediate drug resistance by acting as sponges, other molecular mechanisms have also been reported, such as maintaining the stability of downstream miRNAs [166]. Common mechanisms underlying drug resistance include DNA repair, abnormal activation of signaling pathways, cancer stemness and the EMT [218]. A primary action of chemotherapy is to mediate DNA damage in cells, which in turn induces cancer cell death. In contrast, DNA repair determines the ability of cells to resist drugs. The NER pathway is essential for repairing DNA cross-linking damage caused by chemotherapeutic drugs such as cisplatin [219]. The deletion of circ_0001946 might activate the NER pathway to increase DNA repair, leading to cisplatin resistance in A549/DDP cells [136]. Abnormal activation of oncogenic signaling pathways inhibits drug-mediated cell death by transmitting bypass or downstream signals. AKT, a dominant effector of PI3K signaling, induces cell survival and proliferation by regulating the expression of genes with antiapoptotic activity, such as CREB and NF-kB [220]. CircAMOTL1 increases the levels of the both phosphorylated and total AKT proteins to induce taxol resistance in BC [100]. The Wnt/β-catenin signaling pathway is another classic resistance-related pathway [221]. Its activation by circ_001569 also contributes to cisplatin resistance in OS [142]. Cancer stem cells (CSCs) have been regarded as “tumor-initiating cells”. Since the recognition of cancer heterogeneity, CSCs, a small proportion of cancer cells, were considered to contribute to therapy resistance [222]. Circ_001680 upregulates BMI1, a positive regulator of stem cell-like properties, by targeting miR-340. A flow cytometry-based analysis further confirmed that the circ_001680/miR-340/BMI1 axis increases the CSC population, resulting in metastasis and irinotecan resistance in CRC [59]. The EMT is referred as a type of cellular reprogramming from the epithelial to mesenchymal-like phenotype. Morphological changes have been observed during the EMT process. This change has also become an obstacle to therapy [223]. CircRNAs are also involved in EMT-mediated resistance. CircCRIM1 promotes NPC metastasis and docetaxel resistance by upregulating FOXQ1 to enhance the EMT [111].

**Table 3:** CircRNAs in non-platinum cytotoxic drugs

Cell cycle specificity	Drug	Cancer	CircRNAs	Mechanism	Role	Ref
S phase	Irinotecan	Colorectal Cancer	circ_001680	MiR-340/BMI1 axis	Up	[59]
	Pemetrexed	Gastric Cancer	circMTHFD2	MiR-124/ FDZ5/MDR-1 axis	Up	[66]
	Gemcitabine	Renal Cancer	circ_0035483	MiR-335/CCNB1 axis	Up	[76]
		Pancreatic Cancer	chr14:101402109–101,464,448+; chr4:52729603–52,780,244+;	Downregulate miR-145-5p	Up	[83]
M phase	Taxol	Gastric cancer	circPVT1	MiR-124-3p/ZEB1 axis	Up	[92]
		Ovarian Cancer	circ_0063809	MiR-1252/ FOXR2 axis	Up	[98]
		Breast Cancer	circAMOTL1	Activating AKT pathway	Up	[100]
		NSCLC	circ_0002483	MiR-182-5p/GRB2/FOXO1/FOXO3 axis	Down	[106]
	Docetaxel	Lung Adenocarcinoma	circ_0003998	Downregulate miR-326	Up	[107]
		Nasopharyngeal Carcinoma	circCRIM1	MiR-422a/FOXQ1 axis	Up	[111]
Cell cycle- nonspecific	Doxorubicin	Acute Myeloid Leukemia	circPAN3	MiR-153-5p/miR-183-5p/XIAP axis	Up	[114]
		Breast Cancer	circ_0006528	MiR-7–5p/ Raf1 axis	Up	[117]
			circKDM4C	MiR-548p/ PBLD axis	Down	[122]

*Ref* Reference

**Table 4:** CircRNAs in platinum drugs

Drug	Cancer	CircRNAs	Mechanism	Role	Ref
Oxaliplatin	Colorectal Cancer	circ_032883	Sponge miR-501-5p	Up	[126]
		circ_0338	NA	Up	[127]
		circ_0005963	MiR-122/PKM2 axis	Up	[128]
Cisplatin	NSCLC	circ_0004350; circ_0092857	Synergy effect with the parental EIF3a gene	Up	[131]
		circ_0076305	MiR-296-5p/STAT3 axis	Up	[132]
		circPVT1	MiR-145-5p/ABCC1 axis	Up	[135]
		circ_0001946	Activating the NER signaling pathway to increased DNA repair ability	UP	[136]
	Osteosarcoma	circ_0004674	NA	Up	[140]
		circ_0001258	MiR-744-3p/GSTM2 axis	Up	[141]
		circ_001569	Activating Wnt/β-catenin signaling pathway	Up	[142]
		circPVT1	Upregulate ABCB1	Up	[143]
		circ_0081001	NA	Up	[145]
	Hepatocellular Cancer	circRNA_101505	MiR-103/NOR1 axis	Down	[151]
	Gastric Cancer	circ_0081143	MiR-646/CDK6 axis	Up	[154]
		circAKT3	MiR-198/ PIK3R1 axis	Up	[153]
		circFN1	Sponging miR-182-5p	Up	[155]
	Ovarian Cancer	Cdr1as	MiR-1270/SCAI axis	Down	[156]
	Bladder Cancer	circ_000285	NA	Down	[157]
		circFNTA	MiR-370-3p/FNTA axis	Up	[158]

*Ref* Reference

**Table 5:** CircRNAs in other drugs

Classification	Drug	Cancer	CircRNAs	Mechanism	Role	Ref
Endocrine Drugs	Enzalutamide	Prostate Cancer	circ_0004870	U2AF65/AR-V7 axis	Down	[165]
			circRNA17	Maintaining stability of miR-181c-5p	Down	[166]
			circUCK2	MiR-767-5p/TET1 axis	Down	[167]
	Tamoxifen	Breast Cancer	circ_0025202	MiR-182-5p/FOXO3a axis	Down	[172]
			circBMPR2	MiR-553/USP4 axis	Down	[174]
Targeted drugs	Gefitinib	NSCLC	circ_0109320	NA	Down	[179]
			circ_0004015	MiR-1183/PDPK1 axis	Up	[180]
	Imatinib	Chronic Myeloid Leukemia	circBA9.3	Upregulate the ABL1 and BCRABL1 protein expression	Up	[190]
			circ_100053	NA	Up	[191]
Immune drugs	PD-1 inhibitor	NSCLC	circFGFR1	MiR-381-3p/CXCR4 axis	Up	[199]

*Ref* Reference

Notably, the EMT and CSCs have been recognized as participating in the strong crosstalk between drug resistance and metastasis [224, 225]. Most cancer-related deaths are caused by metastasis, particularly in patients presenting drug resistance and tumor recurrence [224]. Obviously, the circRNA-related drug resistance mechanism not only mediates drug resistance but also promotes metastasis. As another example, the circRNA UCK2 simultaneously inhibits invasion and enzalutamide resistance by upregulating TET1 in prostate cancer [167]. TET1 is an important regulator of both cancer stemness and the EMT [226]. The molecular mechanisms of drug resistance regulated by circRNAs exhibit crosstalk with each other, which also lead to complex crosstalk between various malignant phenotypes, such as drug resistance and metastasis. Our understanding of the molecular mechanisms of circRNAs in tumor resistance is still very superficial. CircRNAs undoubtedly play an important role in anticancer drug resistance, and further studies are urgently needed.

CircRNAs are abundantly expressed and may be specifically expressed in a cell- and tissue-specific manner. They also play important biological roles in tumor resistance through various molecular mechanisms. Therefore, the detection of circRNAs in clinical samples may have implications for clinical diagnosis and treatment; in other words, they have the potential to serve as biomarkers. The unique closed loop structure of circRNAs increases their stability in body fluids and blood [227]. Liquid biopsy, a minimally invasive method for analyzing liquid specimens obtained from patients, has been widely used in cancer biomarker detection. Liquid biopsies of typical biomarkers such as cell-free DNA (cfDNA) can provide effective information on tumor diagnosis and treatment [228]. The unique closed loop structure of circRNAs increases their stability in body fluids; thus, circRNAs are a good biomarker for investigation via liquid biopsies [227]. For example, the level of the circRNA FECR1 is aberrantly increased in serum exosomes of small cell lung cancer (SCLC) patients and is associated with the chemotherapeutic response [229]. As mentioned above, 13 circRNAs overexpressed during the progression of PC to castration-resistant PC were detectable in serum samples from patients with castration-resistant PC [164]. Downregulated serum circ_0000285 indicates cisplatin resistance in bladder cancer [157]. Collectively, circRNAs represent potential biomarkers related to resistance to anticancer drugs in clinical applications. Dynamic monitoring is a promising strategy to comprehensively assess the cancer status and therapeutic response [230]. Notably, the serum circ_0081001 level gradually increases in patients with OS during treatment with cisplatin-based chemotherapy, indicating the potential of circRNAs in dynamic monitoring [145]. With the discovery of increasing numbers of circRNAs with the potential to serve as biomarkers, we must comprehensively consider the sensitivity and specificity of these serum circRNAs for monitoring drug resistance to select the most suitable candidate molecules.

In recent years, the development of effective therapies to regulate ncRNAs has been an active area of investigation. Hundreds of clinical trials involving ncRNAs are already underway [19]. For example, mimics of miR-34, a tumor suppressor miRNA, have reached phase I clinical trials [231]. The most classic components of RNA drugs are oligonucleotides that specifically targeting the ncRNA and transporters used for transport and maintenance of stability. Studies of circRNAs are still in the early stages, and we still lack the technology to safely and effectively regulate circRNAs in the human body. To date, no preclinical trials of circRNAs for cancer treatment have been reported [232]. However, some features of circRNAs make them worth exploring as potential therapeutic targets.

On one hand, circRNAs are potentially useful as direct therapeutic targets by modulating their expression. Some of the main oncogenic circRNAs are abnormally expressed in a variety of tumors and are associated with multidrug resistance. Precise regulation of these oncogenic circRNAs may exert a therapeutic effect on more malignant tumors. The unique back splice junction of circRNAs represents a potentially specific therapeutic target because it distinguishes circRNAs from the linear RNAs transcribed from the same genes. In other words, we will be able to more precisely regulate these oncogenic circRNAs without affecting functions of normal linear RNAs. For example, as described above, circPVT1 is upregulated in gastric cancer, NSCLC and osteosarcoma. This upregulation leads to multidrug resistance, including Taxol, oxaliplatin and cisplatin [92, 135, 143]. Precise circPVT1-specific RNA drugs may have potential applications in treating multidrug resistance in all these tumors. On the other hand, because of their stability and their multiple binding sites for miRNAs, tumor suppressor circRNAs are potential therapeutic sponge vectors [27]. For example, circ_0002483 might sponge miR-182-5p to increase Taxol sensitivity in patients with NSCLC [106]. CircRNA_101505 alleviates cisplatin resistance in HCC by sponging miR-103 [151]. The sponging capacity of tumor suppressor circRNAs might be used to specifically design and target specific oncogenic miRNA profiles, subsequently exerting a therapeutic effect. A major challenge to the implementation of any of the aforementioned strategies will be accurately transport into the malignant cells to avoid side effects on normal cells.

## Conclusion

In summary, circRNAs have been thoroughly proven to participate in anticancer drug resistance. We reviewed the functions of circRNAs and potential mechanisms by which these circRNAs regulate resistance depending on the classification of anticancer drugs. These circRNAs have great potential in monitoring and overcoming anticancer drug resistance, and many circRNAs that are deeply involved in drug resistance remain unknown. Further investigation of these resistance-related circRNAs will expand their clinical potential.

## Data Availability

Not applicable.

## Abbreviations

ncRNA: noncoding RNAs
circRNAs: circular RNAs
EcircRNAs: Exonic circRNAs
ciRNAs: intronic circRNAs
EIciRNAs: Exon-intron circRNAs
CRC: Colorectal cancer
5-FU: 5-Fluorouracil
PC: Pancreatic cancer
GO: Gene ontology
BC: Breast cancer
NSCLC: Non-small cell lung cancer
3′-UTRs: 3′ untranslated regions
CircInteractome: Circular RNA Interactome
NPC: Nasopharyngeal carcinoma
AML: Acute myeloid leukemia
KEGG: Kyoto Encyclopedia of Genes and Genomes
PBLD: Phenazine biosynthesis-like domain-containing protein
DACH: (1R,2R)-1,2-diaminocyclohexane
circRIP: circRNA immunoprecipitation
CDR1: Cerebellar degeneration-related protein 1
HCR: Host cell reactivation
NER: Nucleotide excision repair
OS: Osteosarcoma
ceRNA: competing endogenous RNA
HCC: Hepatocellular cancer
AR: Androgen receptor
TAM: Tamoxifen
EGFR: Epidermal growth factor receptor
EGFR-TKIs: EGFR tyrosine kinase inhibitors
PFS: Progression-free survival
EGF: Epidermal growth factor
CML: Chronic myeloid leukemia
cfDNA: cell-free DNA
SCLC: Small cell lung cancer
EMT: Epithelial-mesenchymal transition
CSCs: Cancer stem cells
